# Immune Checkpoint Inhibitor-Induced Myositis Causing Severe Ptosis: A Case Report

**DOI:** 10.7759/cureus.73444

**Published:** 2024-11-11

**Authors:** Nadine A Abdeljabbar, Sabrina Genovese, Mohamed B Haradwala, Manjamalai Sivaraman

**Affiliations:** 1 Department of Neurology, University of Missouri School of Medicine, Columbia, USA

**Keywords:** eyelid ptosis, high-dose steroids, immune-checkpoint inhibitor (ici), immune-related adverse events (iraes), keytruda induced myositis, myesthenia gravis, neuromuscular junction disorders, plasma exchange therapy

## Abstract

Pembrolizumab (Keytruda), an immune checkpoint inhibitor, has been increasingly utilized in the treatment of metastatic urothelial carcinoma. While offering a favorable safety profile compared to traditional chemotherapy, it presents unique risks, including immune-related adverse events (irAEs). This case report describes a 77-year-old woman with a history of invasive bladder cancer treated with pembrolizumab who developed severe bilateral ptosis, myositis, and myocarditis. Initially considered to have 3M syndrome, further evaluation indicated a myasthenia gravis mimic.

This report emphasizes the critical need for vigilant monitoring for early signs of irAEs, such as ptosis, myocarditis, and myositis, in patients receiving pembrolizumab. The patient presented with severe ptosis and respiratory difficulties and was successfully treated with high-dose steroids and plasma exchange therapy, leading to overall improvement. This case highlights the diagnostic challenges of differentiating myositis from myasthenia gravis in patients with ICI-induced complications while advocating for an aggressive treatment approach.

## Introduction

Immune checkpoint inhibitors (ICIs), such as pembrolizumab, have revolutionized cancer treatment by significantly improving life expectancy in patients with advanced malignancies, including non-small cell lung cancer, melanoma, urothelial cancers, and head and neck cancers. [1-3] Pembrolizumab is an IgG monoclonal antibody that blocks the programmed cell death-1 (PD-1) receptor from binding to its ligand, programmed death-ligand 1 (PD-L1). Under normal circumstances, this interaction prevents overactivation of the immune system, acting as a "brake" on immune responses that might otherwise attack healthy tissues. By blocking PD-1, pembrolizumab lifts this brake, enhancing the ability of cytotoxic T-cells to detect and destroy cancer cells [4].

While pembrolizumab is effective in treating cancer, its disruption of immune tolerance can lead to immune-related adverse events (irAEs), where the immune system begins attacking healthy tissues. These adverse effects can manifest in various organ systems, including the skin, gastrointestinal tract, endocrine glands, and nervous system. Neurological irAEs are rare but severe and include neuropathies, myopathies, and myasthenia gravis (MG)-like syndromes [5,6]. The incidence of neurological complications with ICIs is approximately 5% in patients receiving monotherapy, with myasthenic syndromes accounting for 0.12-1.16% of cases [6].

The pathophysiology of pembrolizumab-induced myositis involves the activation of cytotoxic T-cells that infiltrate muscle tissues, leading to muscle fiber necrosis and muscle weakness. Normally, PD-1/PD-L1 interactions prevent the immune system from attacking peripheral tissues. However, pembrolizumab disrupts this balance, triggering autoimmune responses. This immune attack can lead to elevated creatine kinase (CK) levels, muscle weakness, and symptoms that can mimic MG. Differentiating between these conditions is critical for appropriate treatment, as misdiagnosis could delay necessary interventions like plasmapheresis (PLEX) or intravenous immunoglobulin (IVIG) [7].

In this report, we present a case of pembrolizumab-induced myositis and ptosis mimicking MG in a 77-year-old woman. The case highlights the challenges of diagnosing irAEs in the context of ICI therapy, the importance of early recognition, and the need for aggressive treatment to improve patient outcomes.

## Case presentation

The patient is a 77-year-old woman with a history of recurrent Bacillus Calmette-Guérin (BCG)-refractory non-muscle invasive bladder cancer with carcinoma in situ. Her bladder cancer was initially treated with BCG therapy in July 2022, followed by fulguration and gemcitabine in June 2023, and a second BCG induction in October 2023. Due to persistent bladder tumors on repeat cystoscopy, she was referred to hematology/oncology for pembrolizumab therapy, which commenced on June 18, 2024, with planned treatments every three weeks.

Two weeks after her first pembrolizumab cycle, the patient developed a sore throat, diffuse muscle pain, ptosis, and headaches. These symptoms progressively worsened, prompting a clinic evaluation where her condition acutely deteriorated, leading to hospital admission. During her stay, she was suspected of having 3M syndrome (myositis, myocarditis, and seronegative myasthenia gravis), although autoimmune panels returned negative. Cardiac MRI revealed gadolinium enhancement consistent with myocarditis, while femur MRI confirmed myositis without myonecrosis.

**Figure 1:**
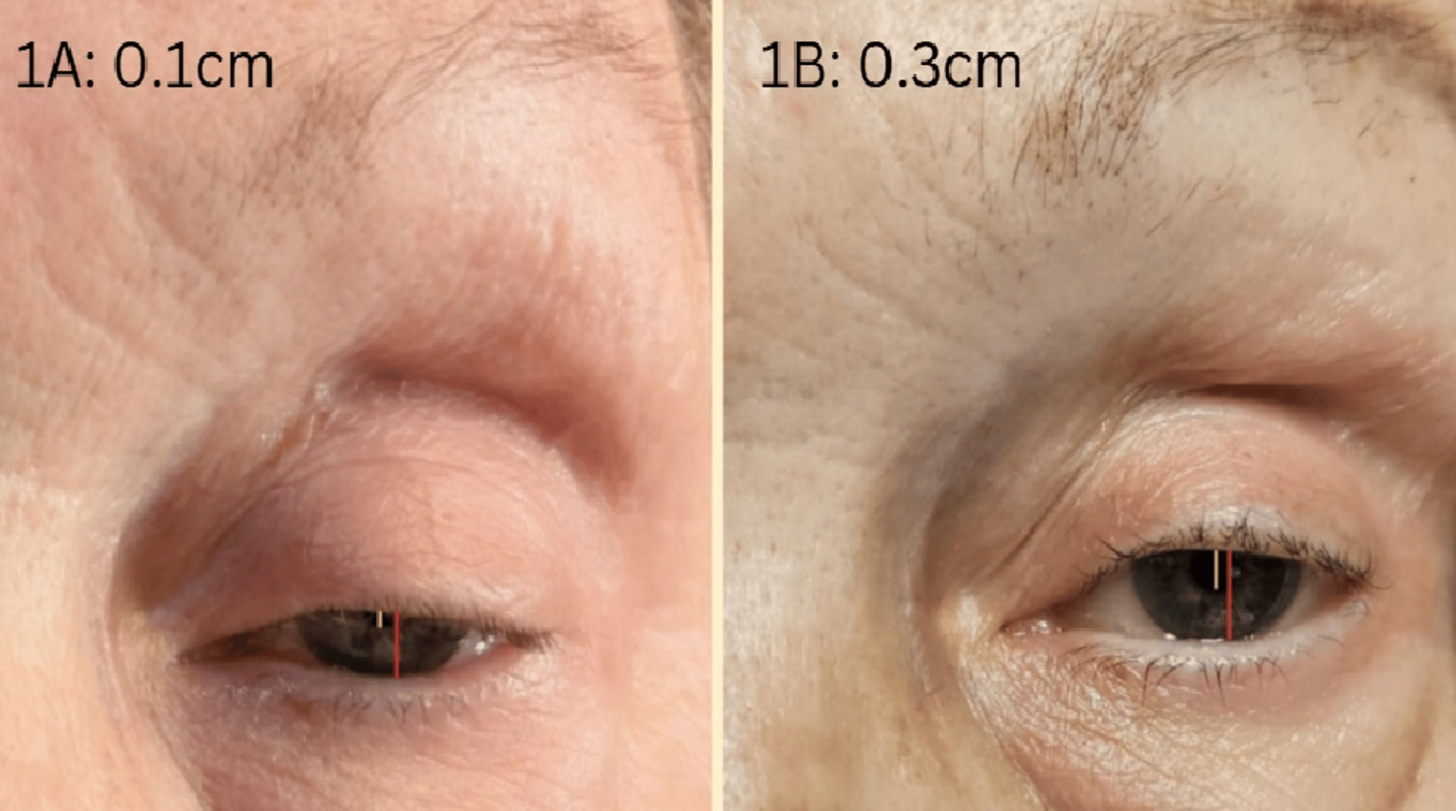
Figure 1A: Ptosis before PLEX treatment, showing a marginal reflex distance 1 (MRD) of 0.1 cm (yellow) and palpebral fissure line (red). MRD is the distance from the margin of the upper lid to the central corneal reflex. A normal MRD1 is between 0.4 and 0.45 cm. Figure 1B: Improvement in ptosis after three PLEX sessions, showing MRD1 improvement to 0.3 cm. [8]

On July 30, 2024, the patient returned to the emergency department with worsening generalized weakness and ptosis. On examination, she exhibited significant bilateral ptosis, more pronounced on the right, with an initial marginal reflex distance 1 (MRD1) of 0.1 cm (Figure 1A). Motor strength was 4/5 in both arms and 4+/5 in both legs for flexion and extension. Cranial nerve function, sensory examination, and reflexes were intact. Provocative tests for myasthenia gravis, including arm abduction for 30 seconds, counting aloud for 20 seconds on one breath, and holding an upward gaze for 20 seconds, showed no signs of fatigue.

The patient’s CK levels peaked at 5294 U/L, significantly above the normal range of 30-200 U/L, indicating widespread muscle damage consistent with myositis. Her troponin I levels were elevated at 6689 ng/mL, suggesting concurrent myocarditis. Following treatment with high-dose steroids and plasma exchange therapy (PLEX), CK levels dropped to 438 U/L, and troponin I decreased to 36 ng/mL. These changes correlated to a clinical improvement noted after her third PLEX session, i.e., her MRD1 improved from 0.1 cm to 0.3 cm (Figure 1B). Ptosis severity is quantified by measuring the marginal reflex distance 1 (MRD1), which calculates the distance between the upper eyelid margin and the pupil. A normal MRD1 is between 0.4 and 0.45 cm [7]. The fifth PLEX session was discontinued due to the development of a deep vein thrombosis (DVT) at the left internal jugular central line site. Given her clinical improvement, the decision was made not to proceed with the final PLEX session.

Treatment strategy and therapy comparison

The patient's rapidly progressing myositis and ptosis prompted the decision to initiate plasma exchange therapy (PLEX). Prior to the initiation of PLEX therapy, the patient was treated with methylprednisolone 1g/day for 2 days since PLEX requires consultation with nephrology at our institution and also the placement of a central line. Table 1 discusses the different modes that can be considered. Theoretically, PLEX can remove IVIG, and therefore we chose to start our patient on PLEX and considered doing IVIG later if needed. Rituximab, though effective in refractory cases, was not considered initially due to its delayed onset of action [2] as well as difficulty obtaining it in an inpatient setting.

**Table 1:** Different immunomodulators, their mechanisms of action, and relevant side effects. IgA: Immunoglobulin A, IL-6: Interleukin 6, Th17: T helper cell 17

Modality of Treatment	Mechanism of Action	Relevant Side Effects
Corticosteroids	Inhibit Lymphocyte, Monocyte, histamine, and complement.	Hyperglycemia, delirium. Long term use can cause osteoporosis. (10.1212/CPJ.0000000000200306)
Intravenous Immunoglobulin (IVIG)	Neutralizes antibodies and inhibits activated complement.	Anaphylaxis in IgA deficiency, thromboembolic events, renal injury. (10.1212/CPJ.0000000000200306)
Plasma exchange (PLEX)	Removes large molecular weight particles including cytokines, antibodies, and inflammatory mediators.	Hypotension, sepsis due to central catheter. (10.1212/CPJ.0000000000200306)
Rituximab	Depletes naïve and memory b-cells	Infusion reactions, anaphylactic reaction, reduces humoral response to vaccines, and can increase risk of infection. (10.1212/CPJ.0000000000200306)
Tocilizumab Satralizumab	Block IL-6 receptors, indirectly inhibiting B-cell, cytotoxic T-cell, and Th17 cell differentiation. doi:10.7759/cureus.49007	Infection, neutropenia.

Following treatment with high-dose steroids and four sessions of PLEX, the patient’s CK improved (5294U/L to 438U/L), and troponin I levels showed marked improvement (6689 ng/ml to 36 ng/ml), correlating with clinical improvements in ptosis and muscle strength. The failure to normalize CK levels after initial steroid therapy had further justified the initiation of PLEX. Due to the development of a deep vein thrombosis (DVT) at the left internal jugular central line site after the fourth session, the final PLEX session was discontinued as the patient demonstrated significant improvement and stabilization of clinical and biochemical markers.

## Discussion

ICIs like pembrolizumab enhance cytotoxic T-cell activity, which can lead to autoimmune-mediated side effects. Differentiating oculobulbar myositis from MG is crucial for management, as myositis typically causes stable weakness, while MG is characterized by fatigue [9]. MG can be worsened by steroids, and therefore, we quickly transitioned to the use of PLEX in our case [1,10]. We present a case of severe bilateral ptosis and fatigue with negative MG provocative testing.

Pembrolizumab-induced myositis has been previously reported with progressive ptosis as the main symptom, similar to our patient [11,12]. Due to the life-threatening nature of these immune-related events, 3M syndrome (myositis, myocarditis, myasthenia gravis) was initially suspected. 3M syndrome includes all three conditions and requires their presence for diagnosis [13]. Our patient did not demonstrate the fatigable weakness typical of MG, nor did she have positive AChR antibody results.

This case demonstrates pembrolizumab-induced levator palpebrae superioris and ocular myositis mimicking Myasthenia gravis, rapidly improving with plasma exchange therapy (PLEX). Before PLEX, our patient had severe bilateral ptosis with an MRD1 of 0.1 cm, indicating profound levator muscle weakness. With PLEX therapy, the patient had improvement in clinical symptoms as well as a decrease in CK and troponin.

Our patient exhibited rapid improvement in ptosis and muscle strength following four sessions of PLEX. This emphasizes the efficacy of PLEX in rapidly progressing myositis cases, particularly when other treatments may take longer to show clinical benefits. The decision to discontinue the fifth PLEX session due to DVT suggests the importance of balancing therapeutic aggressiveness with patient safety in high-risk populations.

Our patient presented with severe bilateral ptosis and elevated CK levels (5294 U/L), with rapid symptom progression leading to PLEX initiation. After three PLEX sessions, significant improvement in MRD1 (0.1 cm to 0.3 cm) and muscle strength was noted. Quantifying improvements in CK levels and MRD1 is critical in assessing treatment efficacy. Our patient’s CK dropped from 5294 U/L to 438 U/L, with concurrent MRD1 improvement, reinforcing the importance of aggressive therapy in life-threatening immune-related adverse events.

Although the patient showed marked improvement following treatment, ongoing follow-up is essential due to the potential for recurrence of irAEs. Monitoring of CK and troponin levels, along with regular clinical evaluations, will be necessary to ensure that the patient does not experience a relapse of myositis or myocarditis.

## Conclusions

This case highlights a novel cause of striking bilateral ptosis in a patient treated with pembrolizumab. Differentiating 3M syndrome from pembrolizumab-induced myositis can impact prognosis. Our case demonstrated improvement with PLEX, underscoring the value of early, aggressive intervention.

As the use of ICIs expands in oncology, understanding the broad spectrum of potential side effects is crucial. Given the severity of its side effects, preventive screening for MG antibodies may help identify patients at risk for neuromuscular complications. As such, we propose that patients should be evaluated for myasthenia gravis symptoms and, if indicated, tested for antibodies.
